# Ceramic Hollow Fibre Constructs for Continuous Perfusion and Cell Harvest from 3D Hematopoietic Organoids

**DOI:** 10.1155/2018/6230214

**Published:** 2018-04-02

**Authors:** Mark C. Allenby, Asma Tahlawi, José C. F. Morais, Kang Li, Nicki Panoskaltsis, Athanasios Mantalaris

**Affiliations:** ^1^Biological Systems Engineering Laboratory, Department of Chemical Engineering, Imperial College London, London, UK; ^2^Transport & Separation Laboratory, Department of Chemical Engineering, Imperial College London, London, UK; ^3^Biological Systems Engineering Laboratory, Department of Hematology, Imperial College London, London, UK

## Abstract

Tissue vasculature efficiently distributes nutrients, removes metabolites, and possesses selective cellular permeability for tissue growth and function. Engineered tissue models have been limited by small volumes, low cell densities, and invasive cell extraction due to ineffective nutrient diffusion and cell-biomaterial attachment. Herein, we describe the fabrication and testing of ceramic hollow fibre membranes (HFs) able to separate red blood cells (RBCs) and mononuclear cells (MNCs) and be incorporated into 3D tissue models to improve nutrient and metabolite exchange. These HFs filtered RBCs from human umbilical cord blood (CB) suspensions of 20% RBCs to produce 90% RBC filtrate suspensions. When incorporated within 5 mL of 3D collagen-coated polyurethane porous scaffold, medium-perfused HFs maintained nontoxic glucose, lactate, pH levels, and higher cell densities over 21 days of culture in comparison to nonperfused 0.125 mL scaffolds. This hollow fibre bioreactor (HFBR) required a smaller per-cell medium requirement and operated at cell densities > 10-fold higher than current 2D methods whilst allowing for continuous cell harvest through HFs. Herein, we propose HFs to improve 3D cell culture nutrient and metabolite diffusion, increase culture volume and cell density, and continuously harvest products for translational cell therapy biomanufacturing protocols.

## 1. Introduction

Cell biomanufacturing platforms for cellular therapy, disease modelling, and tissue regeneration have been limited by nonphysiological cell growth, culture architecture, and ineffective nutrient diffusion to small biomaterial volumes, sparse cell densities, and impure cell product harvests [1]. Culture of human cells in static liquid suspension and 2D systems has been restricted to densities below 5 × 10^6^ cells/mL [2] which improve under enhanced nutrient transfer provided by stirred tank or rocking bioreactors to 10^7^ cells/mL [3]. Higher culture density has been achieved in tissue-mimetic 3D structures of porous scaffolds [4], whereas perfused hollow fibre bioreactors (HFBRs) have reached the highest human cell culture densities reported, nearer that of native tissue (10^8–9^ cells/mL) [5–7]. Despite providing a biomimetic structure and cell density, 3D cultures require termination for cell harvest and are usually mixed with cells of other lineages or maturational stage other than that desired for cell therapy or study [8, 9]. While HFBRs have been applied to continuously extract viral cell products by filtration through hollow fibres (HFs) [10], no fibre has been implemented which can selectively filter cell products for continuous 3D culture biomanufacturing.

Red blood cells (RBCs) represent a cell therapy with high clinical demand: RBCs are required at a rate of 8000 blood units per day in the UK costing 250 million GBP per year [11]. CB-derived RBC production has demonstrated clinical utility for human transfusion [12] but remains limited by unnaturally low production densities and exorbitant medium costs [2]. Physiological blood production takes place within the bone marrow (BM) and is supported by a complex vascular and trabecular architecture to nourish a dense, multilineal, spatially heterogeneous distribution of hematopoietic and stromal cells [13]. The BM produces hundreds of billions of RBCs per day which comprise >95% of peripheral blood cells, but only <25% of marrow cells, due to an efficient *in vivo* filtration [14]. Permeable marrow sinusoids allow for mature cell egress, where maturing reticulocytes deform through tight gaps (1–3 *μ*m) in endothelial cell walls [15, 16], while retaining MNC progeny in the marrow. A 3D porous scaffold integrated with medium-perfused cell-filtering HFs could support tissue-like cell densities, recapitulate physiological cell microenvironments, and continuously harvest RBCs similar to the BM [1, 6, 17, 18].

Herein, we describe the fabrication of dual-layer ceramic HFs consisting of a sponge-filtering layer and a finger-void layer which can be incorporated within 3D porous scaffolds for tissue-mimetic culture systems. These HFs contained a distribution of pore sizes, on average above 0.1 *μ*m, demonstrated an unrestricted filtration of nutrients, metabolites, and cytokines, and appeared able to transport small cells [19, 20]. HFs are investigated for their performance in the filtration of enucleated RBCs from suspensions of CB MNCs and RBCs in three filtration formats: a passive shell-to-lumen filtration (“cross-flow filtration”), a forced lumen-to-shell filtration (“dead-end filtration”), and a cross-flow filtration format adapted for long-term perfused hollow fibre bioreactor culture within a 3D scaffolding (“HFBR culture”). Whereas other studies have fabricated microfluidic devices as effective high-throughput leukoreduction filters [21], we incorporate these ceramic HFs into 3D porous scaffolds serving as a perfusion platform with continuous cell filtration harvest.

## 2. Materials and Methods

### 2.1. Ceramic HF Fabrication and Characterization

Ceramic HFs were fabricated by preparing a mixture of 1 and 4 *μ*m aluminium oxide powders (VWR, Lutterworth, UK) at 58.6% wt/*v* loading density, 1.3% wt/*v* Arlacel P135 (polyethylene glycol 30-dipolyhy-droxystearate; Uniqema, Yorkshire, UK), and 15–30% wt/*v* polyethersulfone (PES) in N-methylpyrrolidone (Sigma-Aldrich, Dorset, UK). This was accomplished over 7–10 days through milling with zirconium balls (Across International, Livingston, NJ) and degassing for 2 hours. The resulting dope solution was extruded through a tube-in orifice spinneret of outer diameter 3 mm and inner diameter 1.2 mm around an inner bore fluid of water or DMSO (Sigma-Aldrich), which fell into a water bath with an air gap of 0 to 15 cm. Eight different HFs were fabricated by adjusting aluminium oxide powder, particle size, PES binder content, type of bore fluid, flow rates of both bore fluid and dope solutions, spinneret-to-water-bath air gap, as well as sintering temperatures on an apparatus previously described [22]. Fibres were first screened for structural integrity and shape and then sintered at high temperatures to form the final products before assessing porosity by mercury intrusion porosimetry (MIP), capillary flow porometry (CFP), and scanning electron microscopy (SEM).

### 2.2. Filtration and Culture Platform Assembly

**Figure 1:**
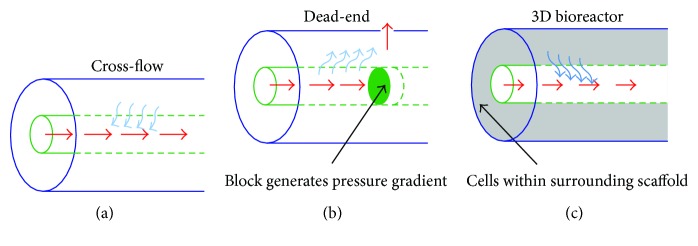
Schematics for hollow fibre cell filtration experiments. (a) Cross-flow filtration: cells are inoculated within the abluminal, shell region, filtered through the fibre into the perfused lumen and are collected at the fibre effluent. (b) Dead-end filtration: a solution of cells is perfused into fibre lumens, which are blocked at the exit with a resin solution so cells must pass through the fibre into the shell and are collected at an opening in the shell. (c) HFBR culture: cells are inoculated into a 3D porous scaffold formed around the fibre, which is perfused for 21 days. Red, blue, and black arrows indicate flow direction, cell movement, and commented regions.

To assess the utility of these ceramic HFs to filter nucleated and enucleated cell fractions isolated from human umbilical cord blood, three different filtration platforms were constructed: (1) a cross-flow filtration platform, (2) a dead-end filtration platform, and (3) a long-term HFBR culture platform (depicted in Figure 1).

In cross-flow filtration (Figure 1(a)), four HFs were adhered within a polyfluoroalkoxy (PFA) fine thread flare tee (Swagelok, London, UK) applying a rapid drying two-component resin (Araldite, Basel, Switzerland) on both the inlet and outlet of the reactor. Medium could then be perfused from the inlet of the reactor, through HF lumens, to the outlet.

In dead-end filtration (Figure 1(b)), a custom-built shell was machined in-house from a PFA rod (ThePlasticShop, Coventry, UK) containing inlet and outlet barbed perfusion caps and shell release caps. A single HF was adhered inside the shell and blocked at the perfusate outlet with resin. Tubing was attached to collect medium flowing out of the shell release cap.

In long-term 3D HFBR culture (Figure 1(c)), a single HF made with water bore fluid was adhered within a cross-flow filtration PFA shell (Swagelok). A solution of 5% polyurethane (PU; Noveon, Brussels, Belgium) in 1,4-dioxane (Sigma-Aldrich) was dissolved overnight at 60°C followed by the injection of 5 mL into the extraluminal shell space and subsequent freezing at −80°C for 2 hours. The 1,4-dioxane was selectively sublimed through thermally induced phase separation (TIPS) by applying a vacuum pressure of 0.01 mbar at −15°C to leave a porous PU scaffold, subsequently coated with bovine collagen type 1 (Sigma-Aldrich) by thoroughly immersing, mixing, and perfusing the HFBR with phosphate-buffered saline (PBS, Life Technologies, Paisley, UK), 70% ethanol (VWR), 62.5 *μ*g/mL bovine collagen type 1 (Sigma-Aldrich) in PBS at a pH of 7.0 followed by a final wash with PBS.

Prior to cell inoculation, all platforms were sterilized by perfusing and washing the shell side with PBS, ethanol (2 hours), and PBS in multiple washes accompanied by UV sterilization. The perfusion bioreactor platform was compared to a static PU scaffold platform in the form of 0.125 mL cubes prepared, coated, and sterilized similarly as previously described [4]. All platforms were then conditioned in cell culture medium for at least 24 hours.

### 2.3. Study Approval

The study has been conducted in accordance with the Declaration of Helsinki and received the required ethics and local research approvals (reference 05/Q0405/20, London-Harrow NRES Committee, UK).

### 2.4. Isolation and Inoculation of CB

MNC fractions were isolated from freshly collected CB (NHS Blood & Transplant, Colindale Blood Bank, UK) by Ficoll-Paque (Sigma-Aldrich) yielding approximately 70–80% MNCs and 20–30% enucleated cells. For filtration experiments, cells were then stored in 90% human serum at 4°C for up to 10 hours and recounted prior to inoculation. CB cell suspensions were inoculated into platforms as enumerated below, and all the systems were perfused at a rate of 20 mL/hour.

- Cross-flow filtration: 3 × 10^7^ cells/mL were seeded into two 5 mL PFA shells and perfused for 24 hours. Filtration surface area: 10–12 cm^2^.
- Dead-end filtration: 5 × 10^6^ cells/mL were seeded into a 480 mL reservoir and perfused into four platforms for 6 hours. Filtration surface area: 0.6–0.7 cm^2^.
- HFBR culture: 4 × 10^7^ cells/mL were seeded into 5 mL of porous scaffolding in one PFA shell and perfused for 21 days. Filtration surface area: 3 cm^2^.

HFBR culture medium consisted of a 60 mL recycled batch of IMDM supplemented with 30% *v*/*v* fetal bovine serum (FBS; Life Technologies), 1% *v*/*v* penicillin–streptomycin (ATCC, Maryland, USA), and 100 ng/mL stem cell factor (SCF; R&D Systems, Abingdon, UK). From day 2, the recycled batch was exchanged for fresh medium with 1 IU/mL erythropoietin (EPO; R&D Systems) and without SCF at a continuous rate of 0.5 mL/h from a 100 mL solution of fed medium replenished weekly. This 5 mL HFBR perfused with a 60 mL reservoir of medium was compared with nonperfused, static, 0.125 mL collagen-coated PU scaffold cubes prepared similarly and cultured in 1.5 mL of supernatant medium exchanged every 2 days.

The number of total, viable, and mononuclear cells was counted using a hemocytometer and trypan blue (StemCell Technologies, Grenoble, France) or methylene blue (StemCell Technologies) dye exclusion stains.

### 2.5. Flow Cytometry

Suspensions of 10^6^ cells/mL were stained with Hoechst 33342 (300 *μ*g/mL, Thermo Fisher) and calcein AM (2.5 *μ*g/mL, Thermo Fisher) in PBS for 45 minutes at room temperature, prepared according to manufacturer's specifications. Suspensions were then washed with cell staining buffer (CSB), described below, and stained with mouse antihuman monoclonal antibodies in 100 *μ*L of CSB and 20 *μ*L of FBS and incubated for 1 hour at 4°C as detailed in Table 1. Suspensions were then washed with CSB and with PBS and filtered prior to the analysis of 50,000 events on a LSRFortessa flow cytometer with FACSDiva software (BD Biosciences). Fluorochrome detection spillover was compensated in comparison with single-stain controls, and positive marker detection was compared with isotype and unstained controls processed using FlowJo version 10.0.8 (Tree Star, Ashland, Oregon).

CSB was prepared by adding 1% wt/*v* bovine serum albumin (BSA; Sigma-Aldrich) and 0.01% wt/*v* NaN_3_ (Sigma-Aldrich) to PBS.

### 2.6. Confocal Microscopy

To assess viability, frozen fibre or reactor sections were immediately incubated at 37°C in 4 *μ*M ethidium homodimer-1 (Invitrogen) and 2 *μ*M calcein AM (Invitrogen) in culture medium for 1 hour followed by washes with PBS and imaging on a Leica SP5 upright confocal microscope with Leica LAS AF software (Leica, Milton Keynes, UK).

To assess cell type, frozen sections were prepared similar as previously reported [23]. Briefly, sections were immediately fixed with a 4% wt/*v* paraformaldehyde solution (Sigma-Aldrich) in PBS for 12 hours, permeabilised with 0.1% *v*/*v* Triton X-100 (Sigma-Aldrich) in confocal microscopy stain buffer (CMSB, described below) for 2 hours, and blocked with 10% *v*/*v* donkey serum (AbCam, Cambridge, UK) in CMSB for 4 hours at 4°C, followed by an overnight incubation with primary antibodies or isotype controls in CSMB at 4°C as detailed in Table 1, a 6-hour incubation with secondary antibodies in CMSB at 4°C, an overnight incubation in 1 : 200 nuclear (DAPI, Life Technologies), and 1 : 1000 plasma membrane (CellMask Red, Life Technologies) counterstain solution in PBS, then storage in a 0.01% wt/*v* NaN_3_ in PBS solution at 4°C. Each step was separated by multiple 15-minute washing steps in appropriate buffer. Sections were then imaged on a Leica SP5 inverted confocal microscope as mentioned above. Acquired images were manipulated within figures by adjusting contrast and brightness of both sample and negative (isotype) controls identically as presented.

CMSB contained 1% wt*/v* BSA, 0.5% *v*/*v* Tween-20, and 0.01% wt/*v* NaN_3_ in PBS. Secondary antibodies include donkey antirat Alexa Fluor 488 and donkey antirabbit Alexa Fluor 555 at 1 : 1000 dilutions (Thermo Fisher).

### 2.7. Scanning Electron Microscopy

Frozen sections were immediately fixed with a 3% *v*/*v* glutaraldehyde solution (Sigma-Aldrich) in Sorenson's buffer (described below) for at least 12 hours. Sections were then washed twice with Sorenson's buffer and postfixed in 1% wt/*v* OsO_4_ (Sigma-Aldrich) in Sorenson's buffer for one hour followed by two more washes in the same buffer. Sections were then dehydrated in an increasing gradient of absolute ethanol diluted with Sorenson's buffer at 0.067 M, pH 7 (50 : 50, 70 : 30, 90 : 10, 95 : 5, and 100 : 0 twice), followed by further drying in an increasing gradient of hexadimethylsilazane (Sigma-Aldrich) in absolute ethanol (70 : 30, 90 : 10, 95 : 5, 100 : 0 twice, Sigma-Aldrich) and left to air-dry overnight. After solvent evaporation, sections were adhered to SEM stubs with carbon tape (Elektron Technology, Stansted, UK), sputter-coated with gold (20 mA, 30 s) prior to imaging on a JSM-6010LA scanning electron microscope (SEM; JOEL, Watchmead, UK). Sorenson's buffer was prepared by titrating solutions of 0.067 M Na_2_HPO_4_ in DI water with 0.067 M KH_2_PO_4_ in DI water (both Sigma-Aldrich) to a pH of 7 directly before use.

### 2.8. Extracellular Metabolite and Protein Analysis

Perfused HFBR medium was collected from 3-way stopcocks (Smith's Medical, Watford, UK) directly before entering and immediately after leaving the HFBR. Extracellular nutrients and metabolites, pH, and dissolved gases were analyzed using a BioProfiler Chemistry Analyzer (Nova Biomedical, Runcorn, UK) and averaged over 3 identical replicates.

## 3. Results

### 3.1. Phase Inversion Bore Fluid Controls Ceramic Fibre Micropore Structure

**Figure 2:**
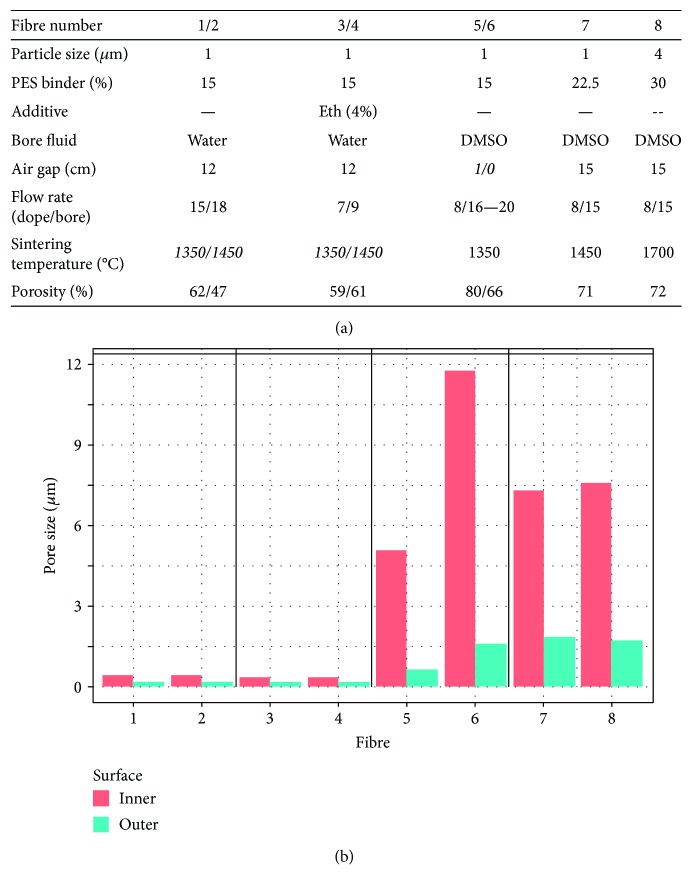
Effect of fabrication parameters on fibre porosity. (a) Table of fibre fabrication parameters producing structural differences in porosity and pore size. Texts in italics identify varied parameters of the fibre/group. (b) Outer and inner surface average pore diameter measured by MIP. Fibres 1 and 5 were defined as the “water fibre” and “DMSO fibre”, respectively.

**Figure 3:**
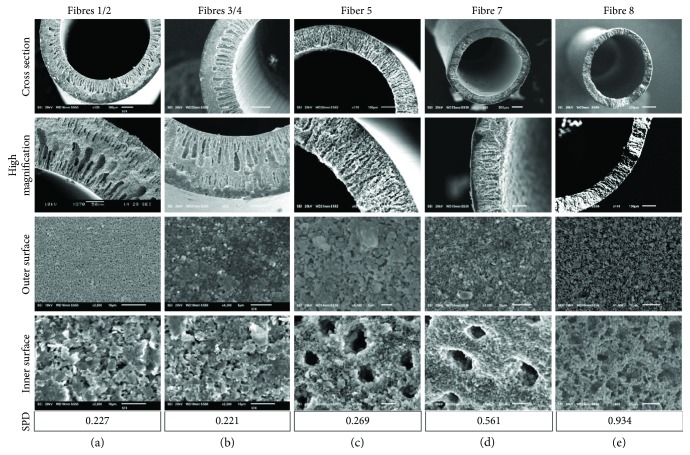
Representative SEM images and smallest pore diameter (SPD) of fabricated fibres. (a) fibres 1 and 2, (b) fibres 3 and 4, (c) fibre 5, (d) fibre 7, and (e) fibre 8. Fibres 1 and 5 were defined as the “water fibre” and “DMSO fibre”, respectively.

Eight different types of ceramic HFs were fabricated through steps of dope preparation, fibre extrusion, phase inversion, and sintering by adjusting dope alumina particle size and PES binder composition, bore fluid, dope and bore fluid flow rate, and sintering temperatures. Dope and bore flow rates were chosen to maintain a stable fibre extrusion; otherwise, these parameters were varied to enlarge average pore sizes along the fibre's outer surface, which represents the cell filtration-limiting pore size, from 0.2 to 2 *μ*m, increase average pore sizes of the inner surface from 0.35 to 11.5 *μ*m, and increase HF porosities from 45 to 80%. Amongst fabrication parameters inspected (Figure 2(a)), choice of bore fluid most significantly influenced fibre porosity, followed by spinneret-to-water-bath air gap distance, while the addition of 4% *v*/*v* ethanol or altering maximal sintering temperature held no measurable differences (Figure 2(b)). Observation of fibre cross sections (Figure 3) demonstrated finger-like voids formed within an otherwise tortuous ceramic sponge whose diameter, length, and frequency also appeared primarily dependent on bore fluid and air gap distance.

The use of a solvent (DMSO) as a bore fluid instead of water produced larger outer surface pore sizes, inner surface pore sizes, and fibre porosity (fibre 1: 0.18 *μ*m, 0.43 *μ*m, 62% versus fibre 5: 0.62 *μ*m, 5.04 *μ*m, 80%). Fibres spun around a DMSO bore fluid contained larger pore sizes and porosity (fibres 5–8: up to 2 *μ*m, 11.77 *μ*m, 80%). The largest pore sizes attained, in fibre 6, resulted in a structurally unstable fibre, which could only be fabricated at large diameters preventing filtration. Fibre structure could be stabilised by increasing PES binder concentration which led to larger outer surface pore sizes (fibre 7: 1.84 *μ*m, 7.28 *μ*m, 71%;). The use of larger particle sizes in fibre 8 increased limiting pore diameters: from 2.5-fold (fibre 7) to 4-fold (fibre 8) in comparison with fibres 1 and 5 as measured by capillary flow porometry (Figure 3).

From the eight fibres successfully fabricated, four representative fibres (fibres 1, 5, 7, and 8) were selected to be further tested towards cell filtration from the eight fibres successfully fabricated. Fibres 2, 3, and 4 were eliminated due to their similarity to fibres 1, 5, and 7 (Figure 2). Fibre 6 was eliminated due to its large diameter relative to that of the bioreactor shell. However, the pore architecture of fibres 7 and 8 was unable to filter cells and caused platform blockage and rupture for fibre 7 (after 0.5 h) and fibre 8 (after 2 h) during dead-end filtration. Fibre 1 and fibre 5 successfully filtered cells in both platforms and are further described as “water fibres” and “DMSO fibres,” respectively.

### 3.2. Cross-Flow Filtration Enriches Enucleated Cell Fractions

**Figure 4:**
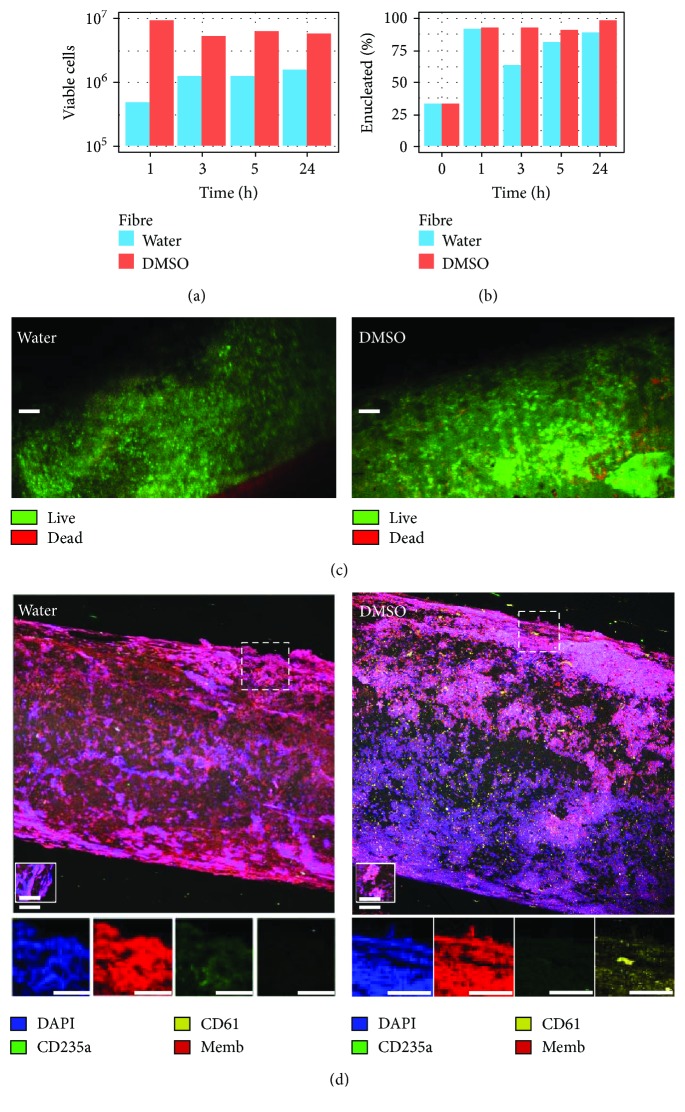
Cross-flow filtration efficiency. (a) Number of viable and (b) percent enucleate cells collected at fibre outlets counted by trypan blue and methylene blue dye exclusion at different time points of filtration. (c) Comparison of cell viability within the water (left) and DMSO (right) fibres after 24 hours of perfusion using confocal microscopy by detection of calcein AM (green), ethidium homodimer-1 (red), and laser reflectance (grey) (100 *μ*m scale). (d) Comparison of cells types remaining within fibres by detection of (top) nuclei (DAPI; blue), red blood cell marker CD235a (green), platelet marker CD61 (yellow), plasma membranes (CellMask; red), and laser reflectance (grey) and (below) single stains of hatched box regions (100 *μ*m scale).

Cross-flow filtration within DMSO fibres occurred at an 8-fold greater rate versus water fibres (Figure 4(a), Table 2). However, DMSO fibres had an 11% lower selectivity for enucleated cells (Figure 4(b)). The number of viable cells filtered through water fibres increased throughout the time points sampled, while DMSO fibres decreased in viable cell numbers collected at successive time points. Filtered cell viability decreased from 90% to almost 60% between 5 and 24 hours of filtration for both fibres, possibly due to HF pore fouling. Cross-flow filtration enriched an inoculate suspension of 30% enucleated RBCs to 92% (water fibre) or 81% (DMSO fibre) enucleated cells in filtrate suspension. Flow cytometry confirmed enucleated CD235a^+^ RBCs were separated from MNCs in cross-flow filtration (Supplementary Figure 1, Table 2).

After 24 hours of filtration, fibres were sectioned for confocal microscopy, and more MNCs were observed within DMSO fibres in comparison with water fibres. Cells which remained inside HFs appeared viable and expressed calcein AM (Figure 4(c)). Less than 1% of cells contained within water fibres expressed CD235a and CD61, whereas DMSO fibres contained a higher number of cells expressing the platelet marker CD61 (Figure 4(d)).

### 3.3. Dead-End Filtration Filters Large Numbers of Enucleated Red Cells

**Figure 5:**
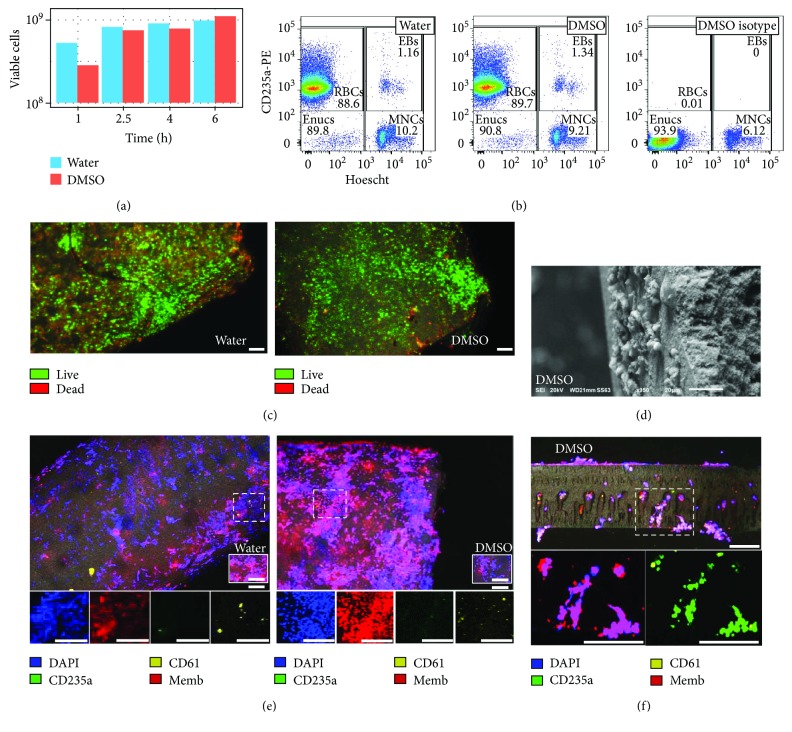
Dead-end filtration efficiency. (a) Viable cell filtrate collection at 1, 2.5, 4, and 6 hours of filtration. (b) Filtrate cell types after 2.5 hours of perfusion for the (left) water and (center) DMSO fibres with (right) DMSO fibre isotype. (c) Comparison of cell viability within the water (left) and DMSO (right) fibre using confocal microscopy with calcein AM (green), ethidium homodimer-1 (red), and laser reflectance (grey) (100 *μ*m scale). (d) SEM of outer, abluminal surface of the DMSO fibre after 6 hours of dead-end filtration (20 *μ*m scale). (e) Comparison of cells remaining within fibres after 6 hours of perfusion within the water (left) and DMSO (right) fibre by confocal microscopy detection of nuclei (DAPI; blue), red blood cell marker CD235a (green), platelet marker CD61 (yellow), plasma membranes (CellMask; red), and laser reflectance (grey) and (below) single stains of hatched box regions (100 *μ*m scale). (f) Confocal images of a magnified traverse section of the DMSO fibre after 6 hours of filtration with (left) identical marker detection and (right) detecting only CD235a (green), CD61 (yellow), and laser reflection (grey) and (below) two stain images of nuclei and plasma membranes or CD235a and CD61 (100 *μ*m scale).

Dead-end cell filtration occurred at least at a 600-fold greater rate versus cross-flow filtration, which was similar for both fibre types (Figure 5(a), Table 2). Dead-end filtration enriched an inoculate suspension of 19% enucleated RBCs to 84% (water fibre) or 81% (DMSO fibre) enucleated cells in filtrate suspension, where 98% of enucleated cells expressed red cell CD235a phenotype (Figure 5(b); Table 2). While filtered cell viability remained >97% for the first 4 hours of filtration, the viability of cells filtered by water fibres decreased to 83% at 6 hours while DMSO fibres remained at 99%, in agreement with cells imaged within water fibres (Figure 5(c)).

Fibres imaged at the end of the 6-hour dead-end filtration indicated that a large number of nucleated cells remained within both water and DMSO fibre types. The viability of cells imaged in the process of dead-end filtration was noticeably less than cross-flow filtration, especially for water fibres, with a higher expression of ethidium homodimer-1 (Figure 5(c)). For both fibres, small cells could be imaged on the abluminal fibre surface after filtration (Figure 5(d)). While a greater number of cells in cross-flow filtration remained within DMSO fibres, dead-end filtration promoted a similar filtration rate for both fibres (Figure 5(e)), and CD235a^+^ enucleated and nucleated erythroblasts could be found in the process of filtration through transverse sections of DMSO fibres (Figure 5(f)).

### 3.4. Continuous Fibre Perfusion Increases Nutrient Exchange and Cell Proliferation in a 3D Porous Scaffold

**Figure 6:**
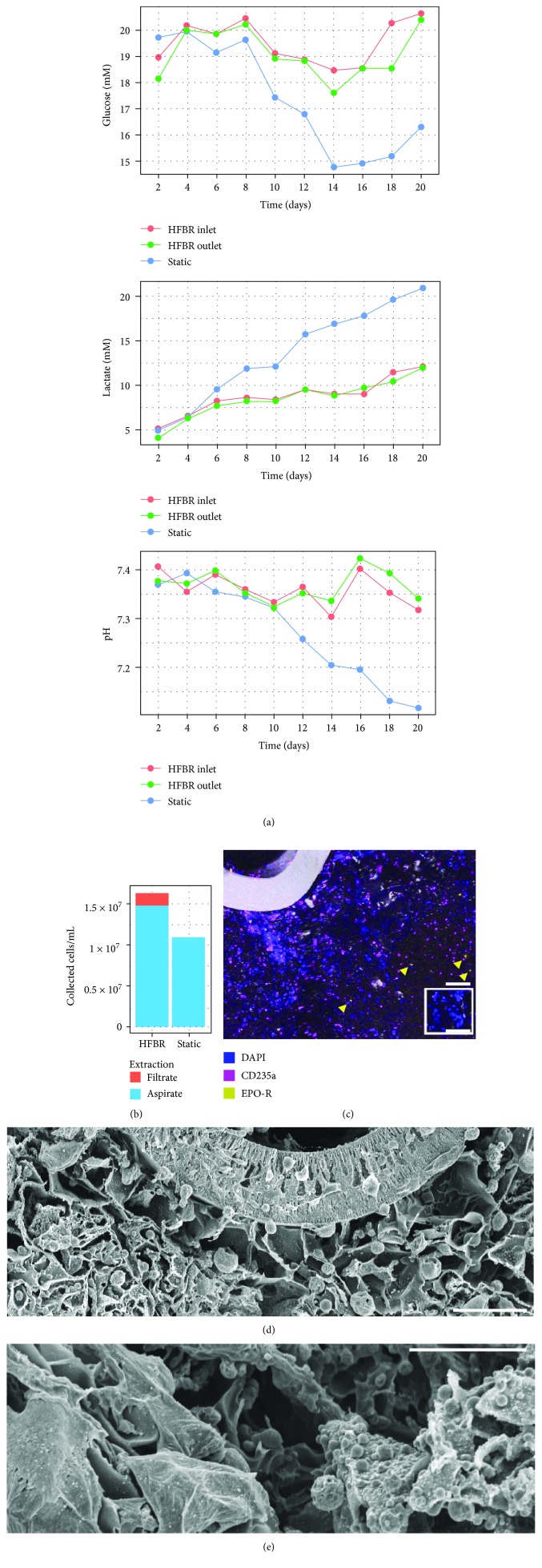
Long-term 3D perfusion hollow fibre bioreactor culture. (a) Measurements of glucose (top), lactate (middle), and pH (bottom) measured at HFBR influent, effluent, and static supernatant every 2 days during culture. (b) Comparison of cells per mL of culture volume extracted at day 21 within perfused HFBR versus unperfused, static scaffold cultures. (c) Confocal microscope image of HFBR cross section at day 21 detecting CD235a (pink), EPO-R (yellow, indicated by yellow arrowheads), nuclei (DAPI; blue), and laser reflection (grey) (100 *μ*m scale bar). SEM images of HFBR cross sections at day 21 of culture highlighting diverse cell morphologies near HFs (d, 200 *μ*m scale bar) or further into the bulk HFBR scaffold (e, 100 *μ*m scale bar).

The perfusion HFBR maintained a more stable extracellular glucose, lactate, and pH profile over 21 days in comparison with that of static cultures, which exhibited toxic pH and lactate levels by the end of culture (pH 7.1, >20 mM lactate; Figure 6(a)). Recycled nutrients and metabolites entering the inlet and outlet of the HFBR varied <5% in concentration, signifying perfusion was rapid enough to approximate pseudo steady-state conditions. The HFBR was replenished with half the amount of medium per inoculated cell versus static culture (1.56 mL versus 3.3 mL over 21 days of culture per 10^6^ CB MNC). While 4 × 10^7^ cells/mL were inoculated, after 21 days only 1.5 × 10^7^ cells/mL could be manually aspirated from the HFBR culture and 10^7^ cells/mL from static culture, while an additional 10^6^ cells were collected within the HFBR-perfused medium (Figure 6(b)).

Mature erythroid phenotype CD235a imaged within the HFBR and static culture comprised 20 and 28% of aspirated cell content, respectively (Figure 6(c)). Cells were observed transgressing the HF (Figure 6(d)) with enucleated RBCs (CD235a^+^DAPI^−^) detected in perfused medium (Figure 6(c)). In situ, HFBR cells comprised small spherical erythroid morphologies in addition to large, spread, adherent morphologies (Figure 6(d)). These cells in situ expressed C-KIT, CD36, EPO-R, CD71, and CD235a phenotypes demonstrating different degrees of erythroid maturation [24], as well as osterix (OSx) and osteopontin (OPN) osteogenic and CD31 endothelial phenotypes known to augment hematopoietic function [25], features of homeostatic and multilineal HFBR erythropoiesis (Supplementary Figure 2).

## 4. Discussion

We have fabricated ceramic HFs that improve nutrient and metabolite diffusion and continuously harvest cell products within millilitre scale 3D porous scaffold cultures. This manuscript studies the effects of different HF fabrication parameters on microscale porosity, CB cell filtration, and enucleated cell separation, and incorporation into a 3D porous scaffold as a HFBR. The major findings suggest the HFs can filter cells at a rate above 10^8^/h whose porosity can be tuned during fabrication to separate different cell types. The HFBR culture demonstrated a more stable metabolic profile despite expanding a greater density of cells within larger volumes than nonperfused 3D cultures.

Two industrially relevant applications exist for further HF development: leukoreduction of blood samples and continuous cell product harvest from long-term 3D hematopoietic cultures. The current clinical standard for leukoreduction is single-use, dead-end filters, which reduce a unit of peripheral blood from 10^8–9^ to 10^6–7^ residual leukocytes, with similar (≥99%) efficiencies being recently achieved by microfiltration platforms [21]. Although the HFs could only reach a 97% dead-end separation efficiency, the filtered suspensions contained a greater ratio of mononuclear to enucleated cells (5 : 1), more relevant to current hematopoietic culture than standard peripheral blood leukoreduction (1 : 1000), where higher MNC contents could lead to fouling. Of these leukoreduction methods, HF filters can be uniquely incorporated within long-term 3D cultures to filter nutrients and cells [19, 20, 26].

Ceramic and predominately polymeric HFs have been characterized in nano- and reverse osmosis filtration platforms for chemical separation [27–30] or in mammalian cell HFBRs to separate viral and antibody products [7, 10, 31–36]. To date, no studies have investigated the role of HFs in the microscale separation of cellular fractions. Herein, we have developed ceramic hollow fibres useful for separating RBCs from CB MNCs suspensions (up to 19% to 90% RBC separation efficiency) at a rate of half a billion cells per hour. Although operating less efficiently than current leukoreduction filters [21], these HFs are able to be integrated into long-term CB 3D porous scaffold bioreactors to improve perfusion and uniquely allow for continuous cell harvest. Nonetheless, HF and HFBR fabrication parameters must be further optimised to limit fouling during filtration and provide viable cell product harvest from HFBR cultures, while the effect of HF medium perfusion on HFBR culture dynamics should be characterized.

### 4.1. Influence of Fabrication Parameters on HF Structure and Filtration

Presented parameters for HF fabrication imparted a wide range of pore sizes, structures, and filtration efficiencies, and parameters effecting mechanisms of spinning and phase inversion seemed especially sensitive [36]. During spinning, bore fluid penetrated the HF membrane, forming large finger-like voids in the ceramic structure which stopped upon contact with the water bath during phase inversion. Therefore, altering bore fluid or time-of-flight between spinneret and water bath (air gap distance) produced longer finger-like void pores: reducing tortuosity but also structural integrity of the fibre.

Spinning fibres around a DMSO bore fluid produced average outer limiting pore sizes between 1-2 *μ*m, closer to those found during reticulocyte egress within bone marrow sinus endothelium (2-3 *μ*m; [37]) versus average limiting pore sizes of fibres spun using water as a bore fluid (0.2–0.4 *μ*m; Figure 2). However, these large pores often rendered fibres structurally unstable to be used (fibre 6) and others contained a tortuous sponge layer without finger-like voids, occluding cell passage and causing rupture (fibres 7 and 8). Therefore, stability and tortuosity and not pore size became the critical factor for cell filtration, and cells could only be filtered using fibres that had uniformly smooth finger-like channel voids. Our filtrate yield may be further increased if fibres with larger pore sizes could be fabricated, but a tight control on pore size distributions must be implemented to not dilute filtrate purity.

Altogether, the best pore sizes were produced by spinning fibres around a DMSO bore while less tortuous finger pores were produced around a water bore. Hence, we propose spinning fibres using a mixed bore of water and DMSO in combination with an air gap of 1-2 cm in order to achieve a long finger pore layer and short sponge layer while maintaining a structurally usable fibre. Furthermore, we propose fabricating fibres using the two different particle sizes utilized herein (1 and 4 *μ*m) to increase the average pore size while improving pore homogeneity similar to that achieved by Tan et al. [38]. Once an optimal HF structure is attained, further functionalisation by surface modification and chemical reinforcement could be tested to provide selective cell attraction and prevent cell adhesion and fouling [39, 40].

### 4.2. Achieving Long-Term Filtration

The small average pore sizes of our fibres did not prohibit the passage of cells, but may have limited filtration efficiency especially through fouling. Two centimetres of our ceramic fibres filtered nearly half a billion RBCs per hour despite having an average pore size below 1 *μ*m. Although MIP only provided an average pore size, we visually observed variability in pore sizes during imaging, and it is likely that cells successfully egressed through larger-than-average pore sizes, while cells became trapped and clogged in smaller-than-average pores.

In cross-flow filtration, larger-pore DMSO fibres (fibre 5) filtered a greater number of cells than water fibres (fibre 1) of lower enucleated selectivity. However, DMSO fibre filtration proceeded at a successively slower rate with time, suggesting the greater quantity of larger, nucleated cells which were imaged within DMSO fibres may have become trapped and fouled fibre filtration. In dead-end filtration, larger pores on the inner HF surface may have allowed more cells to enter and, unable to pass through smaller outer surface pores, remain trapped within the fibre wall causing a reduction of in situ imaged viability. While cells could be imaged filtering through fibres after 21 days of HFBR culture, a much smaller number of cells were found in the HFBR filtrate relative to those aspirated, indicating that while small pore sizes did not prohibit the passage of cells, their filtration may have been hampered during long-term culture.

Tangential flow filtration represents a promising filtration format to limit cell fouling predominately experienced along abluminal HF surfaces and to improve long-term cell separation and viability. However, tangential flow filtration is likely to provide poorer short-term yield in comparison with dead-end filtration [41], which was able to filter a suspension of 1 × 10^8^ enucleated cells mixed with 5 × 10^8^ MNCs (the latter being equivalent to one CB MNC unit) within 6 hours while maintaining high (>90%) cell viability. Therefore, dead-end filtration was sufficient for CB cell separation. In addition, tangential flow cell filtration would require cells to be part of the mobile phase, which is not as relevant to HFBR culture as cross-flow filtration.

### 4.3. Benefits of HF Perfusion in Long-Term 3D Culture

Our scaled-up HFBR platform demonstrated HF perfusion improved nutrient diffusion, metabolite removal, and cell expansion at culture termination while allowing continuous cell harvest by filtration throughout culture. The HFBR operated at a 40-fold increased scaffold volume (5 mL perfused HFBR versus 0.125 mL nonperfused cubes) and incorporated only one HF when many more could be incorporated in a similar scaffold volume. Even so, the platform demonstrated the perfusion of one HF reduced toxic metabolite accumulations found in static scaffold culture. These toxic metabolites may have prevented cell expansion in both cultures, demonstrating a greater effect for the static cultures. Nutrient diffusion and removal of metabolite wastes are critical for long-term (21 day) culture, but not for short-term (≤1 day) filtration experiments where an excess of medium is supplied and as cross-flow HF transport of glucose and lactate is unrestricted (each with HF permeabilities of 4 × 10^−5^ cm/s) [19, 20]. Cell density and expansion at day 21 were measured by aspiration and may not account for adherent cells imaged in situ. While RBC phenotype CD235a was expressed in HFBR aspirate and *in situ* imaging, no noticeable expression was found for filtered cells, suggesting HFBR cell content was not packed enough to promote significant RBC egress.

Prior studies utilising the static 3D cultures inoculated 2 × 10^7^ CB MNCs/mL for a 54-fold, 28-day expansion in cytokine-free conditions but resulted in limited (10% to 20%) CD235a maturation by the end of culture [4]. Moreover, in HFBR cultures inoculated 10^9^ CB MNCs/mL within a 10 mL scaffold volume, which was perfused at identical flow rates with a similar medium, a 34-fold at 31-day expansion was obtained producing 10^10^ aspirated cells of which only 5% expressed CD235a [19]. In comparison, current 2D hematopoietic-erythroid massive amplification (HEMA) cultures can attain >10^5^ expansion whereby 90% enucleated CD235a RBCs are produced, suitable for human transfusion [2, 12]. However, such HEMA cultures inoculate rare CB CD34^+^ MNCs which are expanded at densities beneath 5 × 10^6^ cells/mL. HEMA cultures require large medium volumes, abnormally high concentrations of expensive cytokines, and production costs 1-2 orders of magnitude above donated RBC unit costs [2, 20, 26]. A recently described immortalized erythroblast line could mitigate cell source limitations by providing unlimited RBC generation [42], while medium requirements and RBC harvest could be made more economically viable using proposed HFBR bioprocessing strategies, where CD235a enucleation rate would be a critical parameter for future study.

The microenvironment for hematopoietic cell proliferation and differentiation is controlled *in vivo* by nutrient and growth factor distribution from marrow vasculature as well as cell-cell and cell-matrix niche interactions [16, 37, 38]. The utility of *in vitro* cellular microenvironments is becoming better understood through decellularised scaffolds comprised of extracellular matrix proteins [43]. In addition, marrow egress is thought to aid RBC maturation by restructuring membrane proteins [15], as the stress created by sinus blood flow and the tight pore geometries provides mechanical stimuli. Aspects of this process have been mimicked *in vitro* for the mechanistic study and production of other cell types, such as platelets stripped from megakaryocyte cytoskeletons at the sinusoidal wall [44, 45]. Further HFBR development should explore the impact of platform geometry, flow rate, medium composition, and cellular inoculum to form a microenvironment for improved RBC production.

## 5. Conclusion

In summary, microporous ceramic HFs enable continuous filtration of RBCs from CB and can be incorporated within 3D culture platforms to improve nutrient diffusion for increased cellular densities and enable continuous cellular harvest by filtration. Two operation modes using ceramic fibres have been implemented with different objectives: cross-flow filtration for passive RBC separation at higher cell viabilities and dead-end filtration for ultrafast and efficient RBC separation. Incorporation of ceramic HFs into a CB-inoculated, 21-day 3D porous scaffold bioreactor improved nutrient diffusion while using less medium, increased terminal CB densities, and allowed for continuous cell harvest. We propose these HFs as efficient manufacturing tools for more physiologically relevant cell therapy bioprocesses.

## Figures and Tables

**Table 1:** Primary antihuman antibody stains implemented for flow cytometry and confocal microscopy. Including primary antibody host species, conjugation if applicable, a brief description, final stained concentration or dilution, clone (if monoclonal) or AbCam product number (if polyclonal), and whether used in flow cytometry (flow) or confocal microscopy (confocal).

Antibody	Description	Concentration	Clone	Application
Mouse CD235a-PE	Mature red cell	12.5 *μ*L/mL	GA-R2	Flow
Mouse CD61-APC	Platelet	42 *μ*L/mL	VI-PL2	Flow
Mouse IgG2b-PE	CD235a Iso	12.5 *μ*L/mL	27-35	Flow
Mouse IgG1-APC	CD61 Iso	42 *μ*L/mL	MOPC-21	Flow
Mouse CD235a-PE	Mature red cell	2.5 *μ*g/mL	GA-R2	Confocal
Rat CD235a	Mature red cell	1.7 *μ*g/mL	YTH89.1	Confocal
Mouse EPO-R	Erythroblast	33 *μ*g/mL	MM-0031-6G7	Confocal
Mouse CD36-APC	Erythroblast	42 *μ*L/mL	CB38	Confocal
Rabbit CD61	Platelet	8.4 *μ*g/mL	EPR2417Y	Confocal
Rabbit CD71	Late erythroblast	8.3 *μ*g/mL	Poly; ab84036	Confocal
Rabbit OSx	Preosteoblast	8.3 *μ*g/mL	Poly; ab94744	Confocal
Mouse OPN	Osteoblast	3.3 *μ*g/mL	53	Confocal
Mouse CD31	Endothelial	33 *μ*L/mL	JC/70A	Confocal
Rat IgG2b	CD235a Iso	1.7 *μ*g/mL	RTK4530	Confocal
Rabbit IgG	CD61/CD71 Iso	8.3 *μ*g/mL	EPR25A	Confocal
Mouse IgG	OPN/CD31 Iso	33 *μ*g/mL	Poly; ab37355	Confocal
Mouse IgG2b-PE	CD235a Iso	2.5 *μ*g/mL	27-35	Confocal
Mouse IgM-APC	CD61 Iso	42 *μ*L/mL	G155-228	Confocal

PE: phycoerythrin; APC: allophycocyanin; EPO-R: EPO-receptor; OSx: osterix; OPN: osteopontin; Iso: isotype; Poly: polyclonal.

**Table 2:** Filtrate collected within 6 hours of cross-flow and dead-end filtration for water and DMSO fibre types. Filtration rate, viability, and enucleated fraction were assessed by manual cell counting on a hemocytometer using trypan blue or methylene blue dye exclusion. Reticulocyte purity was measured by flow cytometry and corresponded to a calcein AM-positive, Hoechst-negative, and CD235a-positive fraction.

Platform	Cross-filtration		Dead-end filtration	
Fibre	Water	DMSO	Water	DMSO
Filtration rate (cells/h)	7 × 10^5^	44 × 10^5^	1.6 × 10^8^	1.8 × 10^8^
*F*_*r*_ per fibre surface area (cells/h × cm^2^)	6 × 10^4^	45 × 10^4^	24 × 10^7^	27 × 10^7^
Cell viability (%)	88	89	96	99
Enucleate purity (%)	92	81	84	81
Reticulocyte purity (%)	1	9	74	71

## Supplementary Materials

Supplementary Materials Supplementary Figure 1: cross-flow filtration MNC retainment. Supplementary Figure 2: expression of hematopoietic, osteogeneic, and endothelial markers within the scaffold region of day 21 HFBR cross sections.
