# Supplementary material for: Ceramic Hollow Fibre Constructs for Continuous Perfusion and Cell Harvest from 3D Hematopoietic Organoids
Source: Stem Cells Int. 2018 Apr 2;2018:6230214. doi: 10.1155/2018/6230214 (PMC5901824; doi:10.1155/2018/6230214)

Supplementary Figure Legends:

Supplementary Figure 1: Cross-flow filtration MNC retainment. (A) Calcein AM-positive pre-gated cells were then analysed for CD235a-PE and Hoescht expression. (B) Inoculated hUCB phenotype pre-filtration with isotype. (C) Cell types remaining in the shell-side after 24 hours for water (top) and DMSO (middle) fibres with DMSO fibre filtrate isotype (bottom). Legend: RBCs – Red Blood Cells; EBs – Erythroblasts; Enucs – Enucleated Reticulocytes; MNCs – Mononuclear Cells.

Supplementary Figure 2: Expression of hematopoietic, osteogeneic, and endothelial markers within the scaffold region of day 21 HFBR cross-sections. (A) A 3D confocal image highlighting the inset cropped for Figure 6C (500 µm scale bar). (B) A large scale 3D confocal image (500 µm scale bar) of demonstrating inset volumes represented in (C) with erythroid, hematopoietic, osteogeneic, and endothelial phenotype markers expressed on nucleated cells within HFBR sections at day 21 (100 µm scale bars), with the bottom image representing a negative isotype control of a static scaffold at day 21. Laser reflection was captured in grey on all images to demonstrate fibre and scaffold structure.

Supplementary Figure 1:

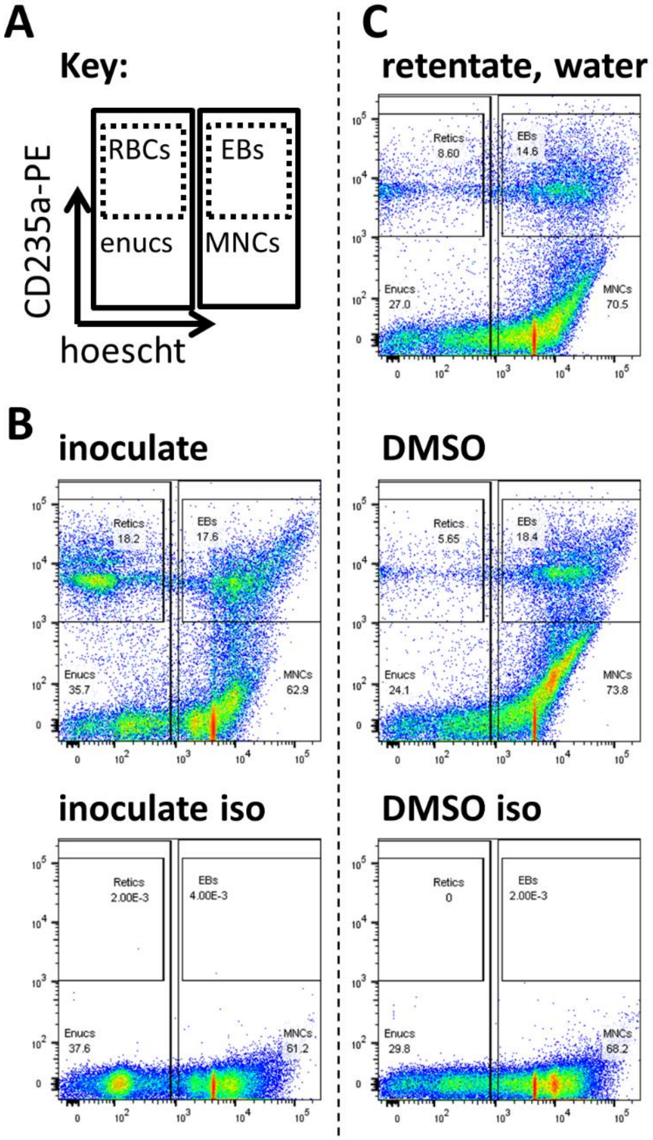

Supplementary Figure 2:

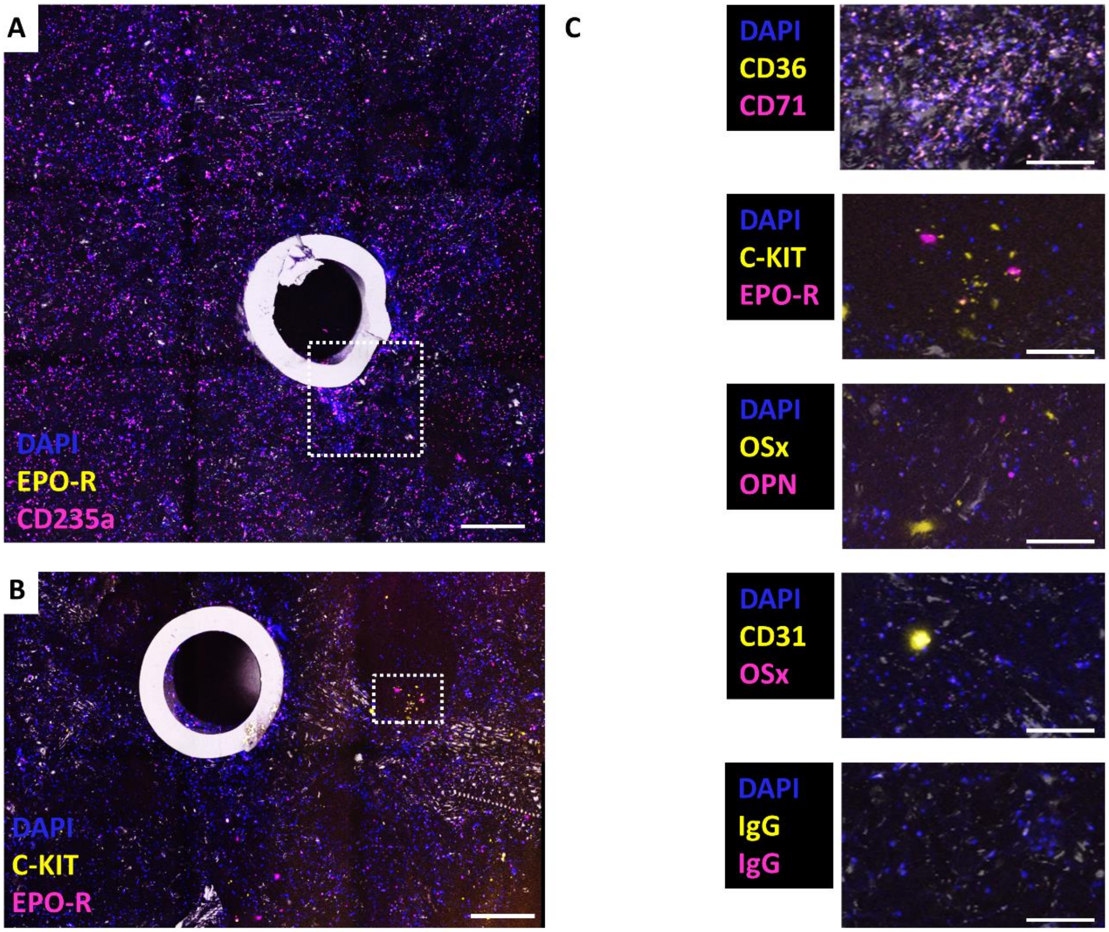

Supplement: Supplementary Materials — Supplementary Figure 1: cross-flow filtration MNC retainment. Supplementary Figure 2: expression of hematopoietic, osteogeneic, and endothelial markers within the scaffold region of day 21 HFBR cross sections. [file 6230214.f1.pdf]
